# Acute Stroke in a Young Patient With Coronavirus Disease 2019 in the Presence of Patent Foramen Ovale

**DOI:** 10.7759/cureus.10233

**Published:** 2020-09-03

**Authors:** Muddasir Ashraf, Sulaiman Sajed

**Affiliations:** 1 Hospital Medicine, UnityPoint Health Trinity Rock Island, Rock Island, USA; 2 Medical Science, Boston University, Boston, USA

**Keywords:** stroke, coronavirus, patent foramen ovale, pfo, young, hypercoagulability, sars-cov-2, covid-19

## Abstract

We present an interesting case of acute ischemic stroke in a 26-year-old patient with coronavirus disease 2019, who presented to the hospital initially with headache, vomiting, and right-sided numbness and tingling. The initial workup was negative, including computed tomography (CT) of the head without contrast and CT angiography of the head and neck with no acute abnormalities. The patient was diagnosed with migraine and discharged from the emergency department. The patient developed worsening symptoms at home in the form of increasing right-sided dysmetria and weakness, gait ataxia, and dysarthria, prompting her to return to the emergency room. Magnetic resonance imaging of the brain was performed and was significant for right-sided acute ischemic cerebellar stroke, with also the involvement of the right cerebellar peduncle. Echocardiogram with a bubble study demonstrated patent foramen ovale. The patient was treated with standard guidelines for stroke.

## Introduction

Novel coronavirus was identified in 2019, and it rapidly reached pandemic proportions. The World Health Organization designated the disease caused by the severe acute respiratory syndrome coronavirus 2 (SARS-CoV-2) as coronavirus disease 2019 (COVID-19). There have been concerns that the virus causes hypercoagulability that leads to thromboembolic events. The pathogenesis of this tendency remains incompletely understood. One such consequence is the increased risk of stroke. Unusually large numbers of stroke cases have been reported worldwide in young patients [1,2], reinforcing our belief that COVID-19 is a systemic disease that affects not only the lungs but also other vital organs. Patients with an inherently increased risk of stroke such as patients with patent foramen ovale (PFO) or previous history of stroke, may be at higher risk of stroke with COVID-19. Hence, the optimal management of these patients needs to be defined in terms of any necessary prophylaxis and treatment.

## Case presentation

A 26-year-old female patient with a past medical history of obesity, post-traumatic stress disorder, depression, and exercise-induced asthma presented to the emergency department with sudden onset of headache, vomiting, and right-sided numbness, and tingling. The patient underwent computed tomography (CT) angiography of the head and neck, which were unremarkable, and no vertebral artery dissection was seen. She was discharged with the diagnosis of migraine.

**Figure 1:**
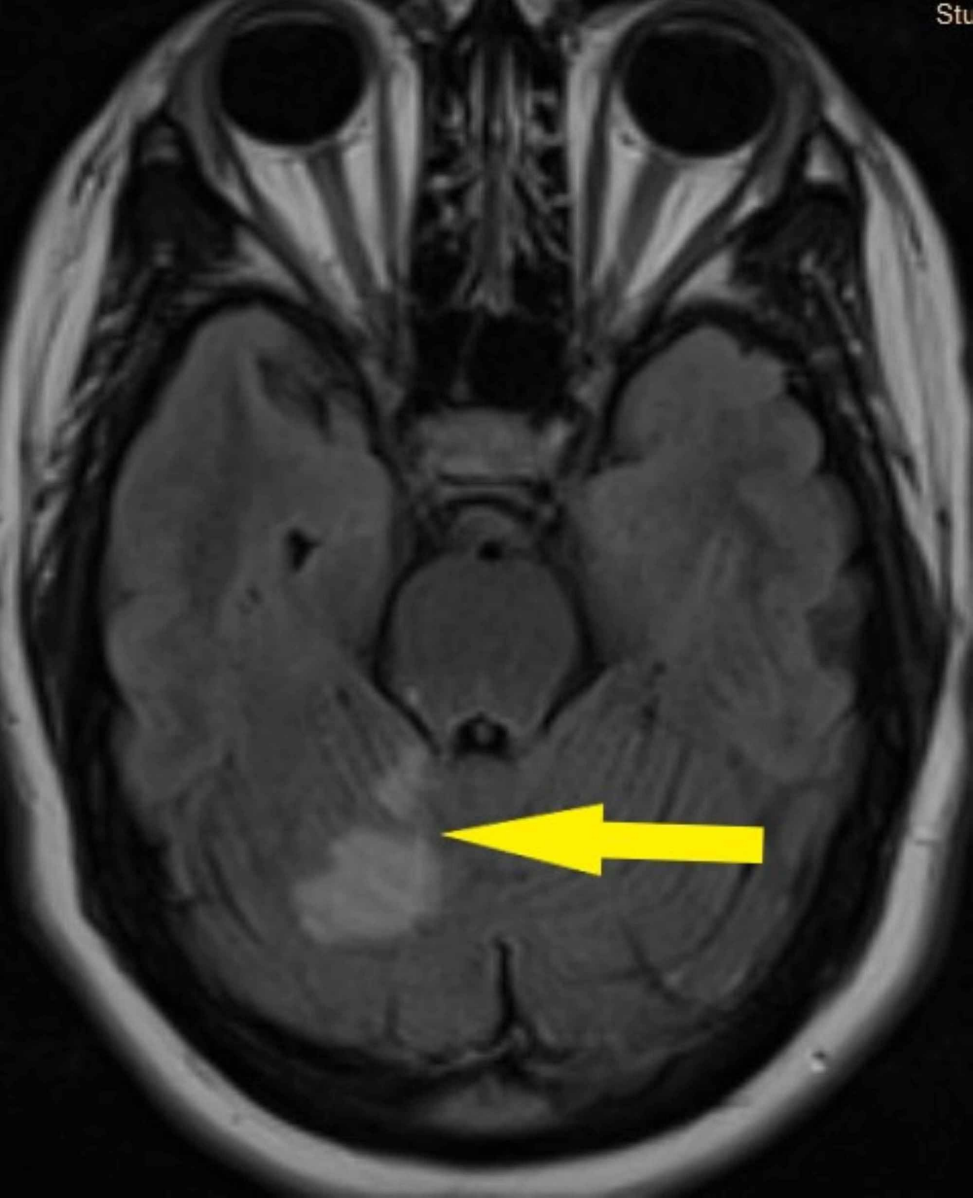
Magnetic resonance imaging of the brain (Flair) shows acute right cerebellar infarct

**Figure 2:**
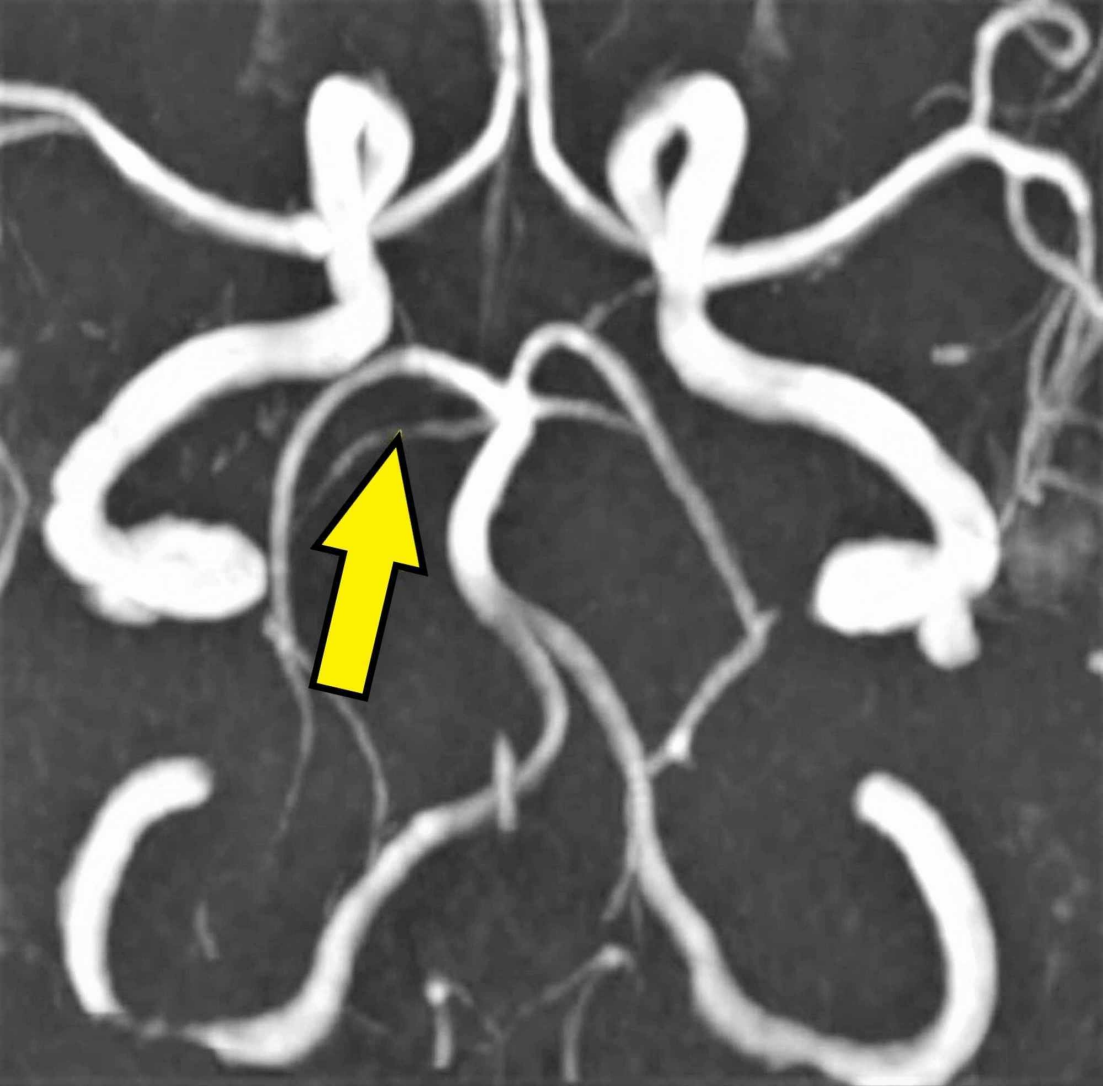
Magnetic resonance angiography of the brain shows narrowing of right superior cerebellar artery

She developed worsening of her symptoms at home, presenting again to the emergency department with increasing right-sided numbness, weakness, and dysarthria. Vital signs were stable with no fever. The patient reported no respiratory symptoms and denied any recent head trauma or chiropractic neck manipulation. The neurological exam was significant for right-sided weakness, dysarthria, right-sided dysmetria, and gait ataxia. Chest X-ray showed no acute infiltrates. Magnetic resonance imaging (MRI) of the brain showed an acute right cerebellar infarct with the involvement of the right cerebellar peduncle (Figure 1). Magnetic resonance angiography of the head revealed narrowing of the right superior cerebellar artery (Figure 2). An echocardiogram with a bubble study was performed, as well as a part of a stroke workup, which showed a PFO. The lower extremity venous doppler ultrasound was negative for deep venous thrombosis. The laboratory test results are shown in Table 1.

**Table 1:** Laboratory investigations WBC, white blood cell; RBC, red blood cell; HGB, hemoglobin; PT, prothrombin time; INR, international normalized ratio; PTT, partial thromboplastin time; APTT, activated partial thromboplastin time; DRVVT, dilute Russell viper venom time; LDL, low-density lipoprotein

Analyte	Ref Range and Units	Results
WBC count	3.60-11.00 10*3/µL	12.06
RBC count	3.92-5.13 10*6/µL	4.16
HGB	11.6-15.0 g/dL	11.7
Platelets	130-450 10*3/µL	304
PT	11.6-14.4 S	13.3
INR	0.9-1.1	1.1
PTT	22-34.7 S	27.3
Antithrombin activity	80-130%	110
Protein C activity	70-150%	113
Protein S functional	50-160%	122
Homocysteine	3.2-10.7 µmoles/L	7.2
Lupus PT	9.4-12.5 S	11.4
Lupus PT INR	0.9-1.1	1
Lupus APTT	25-37 S	22
DRVVT screen ratio	<1.2 ratio	1.01
Cardiolipin antibody immunoglobulin A	0-19.9	<0.5
Cardiolipin antibody immunoglobulin G	0-19.9	<1.6
Cardiolipin antibody immunoglobulin M	0-19.9	<0.2
D-Dimer	0-0.49 Ug/mL	1.2
C-reactive protein	0-5 mg/L	30
LDL	<100 mg/dL	87
Ferritin	8-252 ng/mL	500

The patient was screened for COVID-19 with a nasopharyngeal reverse transcription polymerase chain reaction due to increased reports of stroke in young patients nationwide, and the test came back as positive, to our surprise. Standard stroke treatments, including aspirin, clopidogrel, and high-intensity statins, were initiated for the patient. Enoxaparin for deep venous thrombosis prophylaxis was also prescribed.

## Discussion

COVID-19 has been linked to an increased risk of venous thromboembolism and arterial thrombosis, including stroke in case series from different health centers and anecdotal reports. A single health system identified five cases of acute ischemic stroke associated with COVID-19 over two weeks, with symptoms suggesting large-vessel occlusion; all patients were under 50 years of age [1]. Before the pandemic, there were approximately 0.7 large vessel strokes per two-week interval in patients younger than 50 years. In one of the series of intensive care unit (ICU) patients, ischemic stroke was observed in three of 184 (cumulative incidence, 3.7%) [2]. In another one of the series, cerebral ischemia was seen in three of 150 [3]. In the series that included 314 non-ICU inpatients, six (2%) underwent ischemic strokes, and an additional three in the ICU underwent an ischemic stroke [4].

The pathophysiology is not completely clear, but proposed mechanisms include endothelial inflammation, stasis, and increased procoagulant factors in the blood (hypercoagulability), consistent with Virchow's triad. There is evidence of direct invasion of endothelial cells by SARS-CoV-2. Other sources of endothelial injury include intravascular catheters, mediators of acute systemic inflammatory response such as cytokines (e.g., interleukin-6), and other acute-phase reactants [5]. The contribution of complement-mediated endothelial injury has also been suggested [6]. Hyperviscosity was demonstrated in a series of fifteen critically ill patients in the ICU [7]. Highly elevated levels of D-dimer have been noted as well, which correlates with disease activity. Laboratory findings were characterized in a series of 24 selected patients with severe COVID-19 pneumonia (intubated) who were evaluated and also underwent standard coagulation testing and other assays, including von Willebrand factor. The results showed normal or slightly prolonged prothrombin time (PT) and activated partial thromboplastin time (aPTT), normal or increased platelet counts, increased fibrinogen, and increased D-dimer [8]. Early case series, including a series of 183 consecutive patients from Wuhan, China, suggested that thrombocytopenia and prolongation of the PT and aPTT were more marked [9-12].

It is not clear why these results differed somewhat from later findings of less severe PT and aPTT prolongation. One possible explanation is that these patients were sicker, perhaps because earlier in the pandemic, the disease was not recognized as quickly, resulting in delays in patient presentation and treatment. Another explanation for an isolated prolonged aPTT is the presence of a lupus anticoagulant (LA). Two studies have found a high rate of LA in patients with prolonged aPTT (50 of 57 tested individuals (88%) and 31 of 34 tested individuals (91%)) [3,13]. The presence of an LA may lead to an artifactual prolongation of the aPTT but does not reflect an increased bleeding risk; patients with an LA should receive anticoagulation if indicated.

COVID-19 hypercoagulability has been referred by some experts as a disseminated intravascular coagulation (DIC)-like state, but the main difference is that COVID-19 leads to thrombosis, unlike acute decompensated DIC, which leads to bleeding due to consumption of clotting factors. The consumption of clotting factors is not the case with COVID-19, hence the increased levels of fibrinogen and factor VIII activity that are seen in these patients [8]. Our patient had normal fibrinogen, platelet count, and aPTT but elevated D-dimer, c-reactive protein, and ferritin. Our patient also did not have any respiratory symptoms or chest X-ray abnormalities, but CT of the chest would have potentially revealed abnormal lung findings. This was not performed for our patient due to absent respiratory symptoms and to avoid unnecessary staff exposure.

The interesting question that arises from this case is whether young patients with COVID-19 have an increased risk of stroke in the presence of PFO, and should prophylactic anticoagulation be initiated to prevent stroke until these patients recover from their illness? If they present with acute stroke, should they be started on full anticoagulation to prevent recurrence or worsening of their stroke? Prophylaxis is only possible if the patient had previously diagnosed PFO due to prior episodes of a transient ischemic event or stroke or due to some other indications.

We did not make any such decisions without substantial evidence, given the paucity of data and potential for harm. We did start the patient on prophylactic enoxaparin sodium, which is the standard of care in stroke patients admitted to the hospital for deep venous thrombosis prophylaxis. It potentially has some anti-inflammatory properties as well. The number of patients with COVID-19 who require treatment will most likely be increasing in the near future, not decreasing, and additional studies will be required to answer these questions.

## Conclusions

It has been observed that COVID-19 causes an increased incidence of stroke in young patients. Additional data are required to answer questions regarding the optimal management of these patients, especially in the subset of patients with inherently increased risk, such as patients with PFO, previous history of stroke, a recent history of transient ischemic attack, and asymptomatic but significant carotid disease. This case will hopefully lead to these studies and discussions to further advance our knowledge.
